# Supplementary material for: ZSP1601, a novel pan-phosphodiesterase inhibitor for the treatment of NAFLD, A randomized, placebo-controlled phase Ib/IIa trial
Source: Nat Commun. 2023 Oct 12;14:6409. doi: 10.1038/s41467-023-42162-0 (PMC10570369; doi:10.1038/s41467-023-42162-0)
Supplement: Supplementary file 1 — Supplementary Information [file 41467_2023_42162_MOESM1_ESM.pdf]

Supplementary Information

Supplementary Table 1 Changes in secondary efficacy indicators from baseline in placebo and ZSP1601 treatment groups

			Pooled placebo (N=9)	ZSP1601		
				50 mg QD(N=9)	50 mg BID(N=9)	100 mg BID(N=9)
Lipids	TG, mmol/L	LSmean (95%CI)	1.09(-0.60 to 2.78)	-0.18(-1.85 to 1.49)	-0.81(-2.25 to 0.62)	-0.29(-2.21 to 1.64)
		LSmean difference(95%CI)	-	-1.27(-3.38 to 0.84)	-1.90(-4.15 to 0.35)	-1.38(-3.62 to 0.87)
		P value	-	0.3731	0.1216	0.3574
	TC, mmol/L	LSmean (95%CI)	-0.18(-0.63 to 0.27)	-0.34(-0.81 to 0.12)	-0.69(-1.09 to -0.28)	-0.69(-1.17 to -0.21)
		LSmean difference (95%CI)	-	-0.16(-0.75 to 0.42)	-0.51(-1.13 to 0.11)	-0.51(-1.10 to 0.08)
		P value	-	0.8711	0.1403	0.1101
	LDL-C, mmol/L	LSmean (95%CI)	-0.19(-0.63 to 0.25)	0.16(-0.31 to 0.63)	-0.43(-0.81 to -0.04)	-0.27(-0.74 to 0.19)
		LSmean difference (95%CI)	-	0.35(-0.23 to 0.93)	-0.24(-0.84 to 0.36)	-0.09(-0.66 to 0.49)
		P value	-	0.3684	0.7016	0.9776
	HDL-C, mmol/L	LSmean (95%CI)	0.05(-0.12 to 0.21)	-0.07(-0.23 to 0.10)	0.01(-0.13 to 0.15)	0.06(-0.12 to 0.23)
		LSmean difference (95%CI)	-	-0.12(-0.33 to 0.09)	-0.04(-0.26 to 0.17)	0.01(-0.20 to 0.22)
		P value	-	0.4303	0.9552	0.9992
Blood glucose, mmol/L		LSmean (95%CI)	0.22(-0.41 to 0.86)	0.20(-0.44 to 0.83)	0.07(-0.49 to 0.62)	0.01(-0.67 to 0.68)
		LSmean difference (95%CI)	-	-0.03(-0.85 to 0.80)	-0.15(-1.02 to 0.71)	-0.22(-1.06 to 0.62)
		P value	-	0.9998	0.9610	0.8941
Blood insulin, μU/mL		LSmean (95%CI)	49.48(-32.70 to 131.66)	46.81(-35.35 to 128.97)	34.96(-37.00 to 106.92)	35.85(-51.14 to 122.85)
		LSmean difference (95%CI)	-	-2.67(-107.54 to 102.21)	-14.51(-124.34 to 95.31)	-13.62(-121.44 to 94.19)
		P value	-	0.9999	0.9837	0.9857
Non-invasive biomarkers	CAP*, dB/m	LSmean(95%CI)	22.94(-3.80 to 49.69)	6.24(-20.29 to 32.77)	6.95(-16.65 to 30.56)	-14.38(-43.80 to 15.03)
		LSmean difference(95%CI)	-	-16.71(-50.68 to 17.27)	-15.99(-51.12 to 19.13)	-37.33(-73.26 to -1.39)
		P value	-	0.5446	0.6055	0.0395
	LSM*, Kpa	LSmean(95%CI)	1.36(0.16 to 2.56)	0.94(-0.23 to 2.11)	1.42(0.29 to 2.55)	1.17(-0.14 to 2.47)
		LSmean difference(95%CI)	-	-0.42(-1.82 to 0.98)	0.06(-1.40 to 1.51)	-0.19(-1.67 to 1.28)
		P value	-	0.8433	0.9996	0.9839
	FAST score*	LSmean(95%CI)	0.01(-0.10-0.12)	-0.12(-0.23 to -0.01)	-0.08(-0.17 to 0.02)	-0.19(-0.31 to -0.07)

		LSmean difference(95%CI)	-	-0.13(-0.27 to 0.01)	-0.09(-0.23 to 0.05)	-0.20(-0.35 to -0.06)
		P value	-	0.0642	0.3073	0.0037
		mean (SD)	-0.06(0.14)	-0.18(0.11)	-0.10(0.14)	-0.23(0.12)
		Difference between groups , P value	-	0.2106	0.8633	0.0379
	FIB4	LSmean(95%CI)	-0.01(-0.24 to 0.23)	0.08(-0.15 to 0.30)	-0.04(-0.24 to 0.16)	-0.05(-0.29 to 0.19)
		LSmean difference(95%CI)	-	0.08(-0.20 to 0.37)	-0.03(-0.32 to 0.26)	-0.04(-0.33 to 0.25)
		P value	-	0.8574	0.9926	0.9782
	APRI score	LSmean(95%CI)	-0.02(-0.15 to 0.11)	-0.04(-0.17 to 0.09)	-0.10(-0.22 to 0.01)	-0.17(-0.31 to -0.03)
		LSmean difference(95%CI)	-	-0.02(-0.19 to 0.15)	-0.08(-0.25 to 0.09)	-0.15(-0.32 to 0.03)
		P value	-	0.9894	0.6092	0.1238
Inflammatory cytokines	TNF-α, pg/mL	LSmean(95%CI)	-0.01(-0.15 to 0.12)	0.08(-0.05 to 0.21)	-0.01(-0.12 to 0.11)	0.00(-0.15 to 0.14)
		LSmean difference(95%CI)	-	0.09(-0.08 to 0.26)	0.01(-0.16 to 0.18)	0.01(-0.17 to 0.19)
		P value	-	0.4468	0.9988	0.9979
	IL-6, pg/mL	LSmean(95%CI)	0.39(-1.30 to 2.08)	0.87(-0.70 to 2.44)	1.79(0.41 to3.18)	1.05(-0.67 to 2.77)
		LSmean difference(95%CI)	-	0.48(-1.66 to 2.62)	1.40(-0.74 to 3.54)	0.66(-1.41 to 2.73)
		P value	-	0.9288	0.3018	0.8220
	IP-10, pg/mL	LSmean(95%CI)	6.07(-30.13 to 42.28)	7.71(-24.03 to 39.44)	12.08(-15.82 to 39.99)	13.60(-24.24 to 51.43)
		LSmean difference(95%CI)	-	1.63(-45.92 to 49.18)	6.01(-39.32 to 51.33)	7.52(-34.07 to 49.12)
		P value	-	0.9997	0.9836	0.9601
	CK-18, pg/mL	LSmean(95%CI)	-479.64(-825.87 to -133.41)	-391.61(-750.58 to -32.65)	-410.13(-710.42 to -109.85)	-450.11(-821.55 to -78.67)
		LSmean difference(95%CI)	-	88.02(-358.79 to 534.84)	69.50(-395.57 to 534.58)	29.53(-423.61 to 482.66)
		P value	-	0.9493	0.9768	0.9980
Obesity parameters*		Bodyweight, kg	-1.53(1.39)	-0.80(0.97)	-1.26(0.86)	-1.29(1.31)
		BMI, kg/m2	-0.53(0.48)	-0.27(0.36)	-0.46(0.34)	-0.44(0.49)
		Waist circumference,	-1.72(1.88)	-1.27(1.08)	-2.37(2.17)	-1.23(1.70)
		Abdominal circumference, cm	-2.29(2.16)	-1.56(1.39)	-1.71(1.25)	-1.23(1.54)

Note: *Number of subjects to CAP and LSM in ZSP1601 100 mg BID group was eight. Kruskal Walis test was also used for FAST score. LS means of other indicators were obtained from an ANCOVA model with group as a fixed effect , and baseline values as covariates. Obesity parameters showed the absolute change from baseline. Bold fonts denoted statistically significant values. Satistical test used was two-sided.
