# Supplementary material for: ZSP1601, a novel pan-phosphodiesterase inhibitor for the treatment of NAFLD, A randomized, placebo-controlled phase Ib/IIa trial
Source: Nat Commun. 2023 Oct 12;14:6409. doi: 10.1038/s41467-023-42162-0 (PMC10570369; doi:10.1038/s41467-023-42162-0)
Supplement: Supplementary file 3 — Reporting Summary [file 41467_2023_42162_MOESM3_ESM.pdf]

nature portfolio

Corresponding author(s): Xiaoxue Zhu, Junqi Niu and Yanhua Ding

Last updated by author(s): Sep 1, 2023

Reporting Summary

Nature Portfolio wishes to improve the reproducibility of the work that we publish. This form provides structure for consistency and transparency in reporting. For further information on Nature Portfolio policies, see our Editorial Policies and the Editorial Policy Checklist.

Statistics

For all statistical analyses, confirm that the following items are present in the figure legend, table legend, main text, or Methods section.

n/a	Confirmed	
☐	☒	The exact sample size (n) for each experimental group/condition, given as a discrete number and unit of measurement
☐	☒	A statement on whether measurements were taken from distinct samples or whether the same sample was measured repeatedly
☐	☒	The statistical test(s) used AND whether they are one- or two-sided<br>*Only common tests should be described solely by name; describe more complex techniques in the Methods section.*
☐	☒	A description of all covariates tested
☐	☒	A description of any assumptions or corrections, such as tests of normality and adjustment for multiple comparisons
☐	☒	A full description of the statistical parameters including central tendency (e.g. means) or other basic estimates (e.g. regression coefficient) AND variation (e.g. standard deviation) or associated estimates of uncertainty (e.g. confidence intervals)
☐	☒	For null hypothesis testing, the test statistic (e.g. F, t, r) with confidence intervals, effect sizes, degrees of freedom and P value noted<br>*Give P values as exact values whenever suitable.*
☒	☐	For Bayesian analysis, information on the choice of priors and Markov chain Monte Carlo settings
☒	☐	For hierarchical and complex designs, identification of the appropriate level for tests and full reporting of outcomes
☒	☐	Estimates of effect sizes (e.g. Cohen's d, Pearson's r), indicating how they were calculated

Our web collection on statistics for biologists contains articles on many of the points above.

Software and code

Policy information about availability of computer code

Data collection	no software was used
Data analysis	Statistical analysis was conducted using SAS statistical software for Windows, Release V.9.4 (Armonk, NY, USA). Phoenix WinNonlin version 7.0 (Certara, Princeton, NJ, USA) was used for statistical analysis of the pharmacokinetic parameters. Descriptive statistics were presented using the mean and standard deviation, median, and interquartile range. The least square means (LS Means) of liver biochemical parameters and the 95% confidence interval (95% CI), corrected by Tukey's method, were calculated and used for multiple comparisons of the changes from baseline as well as the differences between escalating dose treatment and placebo groups before and at the end of the 28-day treatment. The Wilson method was used to calculate 95% CI. The Newcombe-Wilson Score method was used for calculating confidence intervals of the difference in the proportion of patients achieving ≥ 30% reduction in liver biochemical parameters and LFC from baseline between two groups. An Analysis of Covariance (ANCOVA) model was used for a comparison of means between groups.

For manuscripts utilizing custom algorithms or software that are central to the research but not yet described in published literature, software must be made available to editors and reviewers. We strongly encourage code deposition in a community repository (e.g. GitHub). See the Nature Portfolio guidelines for submitting code & software for further information.

March 2021

Data

Policy information about availability of data

All manuscripts must include a data availability statement. This statement should provide the following information, where applicable:

- Accession codes, unique identifiers, or web links for publicly available datasets
- A description of any restrictions on data availability
- For clinical datasets or third party data, please ensure that the statement adheres to our policy

The data that support the findings of this study are available from the corresponding authors (zhuxiaoxue@jlu.edu.cn, junqiniu@aliyun.com, dingyanh@jlu.edu.cn). All requests for data will be reviewed by the corresponding authors, and responses will be provided within two months. Access to shared data will require a transfer agreement. However, individual participant-level raw data containing confidential or identifiable patient information will not be available because informed consent did not explicitly include this. The raw generated and/or analyzed data during this study period are protected and are not available due to data privacy laws. Source data have been provided with this paper.

Human research participants

Policy information about studies involving human research participants and Sex and Gender in Research.

Reporting on sex and gender	The study results of phase Ia in healthy subjects of the study drug ZSP1601 showed that there was no significant difference between males and females, so the male or female who met protocol was enrolled in this study but the ratio was not considered. The patients involved in this study were males and females.
Population characteristics	Of the 36 enrolled patients, the presence of NASH was confirmed in 2 patients (1 in the placebo group and 1 in the ZSP1601 50 mg BID group) histologically via liver biopsy, resulting in a NAS of 4 and 5, respectively. The remaining 34 patients were diagnosed based on clinical features of NASH. The average age of the placebo group was 36.33 (28-45) years, with 5 males, There were 4 females, with an average height of 165.98cm, an average weight of 81.02kg, and an average BMI of 29.47 kg/m2; The average age of group ZSP1601 was 33.81 years, with 24 males and 3 females, with an average height of 168.99 cm, an average weight of 83.66 kg, and an average BMI of 29.24 kg/m2.
Recruitment	Recruitment advertisement. This is a double-blined trial, the patients met the inclusion criteria were enrolled. There is no bias.
Ethics oversight	This clinical trial has been registered at ClinicalTrials.gov, number NCT04140123.<br>This study and all amendments have been approved by the institutional review board or independent ethics committee of each study center [the First Hospital of Jilin University (Jilin, China), Nafang Hospital, Nanfang Medical University (Guangzhou, China) and Beijing Friendship Hospital, Capital Medical University (Beijing, China); approval numbers: 19Y110-007, NFEC-201909-Y4, 2019-PI-036-04]. All procedures were performed in accordance with the principles of the Declaration of Helsinki and Good Clinical Practice. Prior to the trial entry, all participants provided written informed consent.

Note that full information on the approval of the study protocol must also be provided in the manuscript.

Field-specific reporting

Please select the one below that is the best fit for your research. If you are not sure, read the appropriate sections before making your selection.

[x] Life sciences [ ] Behavioural & social sciences [ ] Ecological, evolutionary & environmental sciences

For a reference copy of the document with all sections, see nature.com/documents/nr-reporting-summary-flat.pdf

Life sciences study design

All studies must disclose on these points even when the disclosure is negative.

Sample size	The sample size of each dose group was designed on the basis of fewer subjects exposed to the drug to obtain more safety, pharmacokinetic profile, and preliminary efficacy results.
Data exclusions	No data were excluded from the analyses.
Replication	All data quality control and double check. At least 2 investigators or statistician verified the reproducibility of the experimental findings. All attempts at replication were successful.
Randomization	The randomization sequence was generated by an independent statistician. A sheet with the information regarding the allocation and identification numbers was placed in an envelope, which was then sealed.
Blinding	the investigators were blinded to group allocation during data collection and analysis.

Reporting for specific materials, systems and methods

We require information from authors about some types of materials, experimental systems and methods used in many studies. Here, indicate whether each material, system or method listed is relevant to your study. If you are not sure if a list item applies to your research, read the appropriate section before selecting a response.

Materials & experimental systems

n/a	Involved in the study
☒	☐ Antibodies
☒	☐ Eukaryotic cell lines
☒	☐ Palaeontology and archaeology
☒	☐ Animals and other organisms
☐	☒ Clinical data
☒	☐ Dual use research of concern

Methods

n/a	Involved in the study
☒	☐ ChIP-seq
☒	☐ Flow cytometry
☒	☐ MRI-based neuroimaging

Clinical data

Policy information about clinical studies

All manuscripts should comply with the ICMJEguidelines for publication of clinical research and a completedCONSORT checklist must be included with all submissions.

Clinical trial registration	ClinicalTrials.gov, number NCT04140123
Study protocol	ClinicalTrials.gov
Data collection	From June 15, 2020 (the first enrolled subject signed and informed) to August 3, 2021 (the end of the visit for the last subject) in the First Hospital of Jilin University (Changchun, Jilin, China) Nafang Hospital, Nanfang Medical University (Guangzhou, China) and Beijing Friendship Hospital, Capital Medical University (Beijing, China).
Outcomes	Given that the drug was first used in NAFLD patients, safety and tolerability are the primary outcomes, while efficacy and pharmacokinetics are the secondary outcomes. Recently, magnetic resonance imaging-derived proton density fat fraction (MRI-PDFF), a non-invasive measurement of the amount of hepatic fat, has been validated as an accurate biomarker of liver steatosis. In previous clinical trials for NASH, MRI-PDFF had been applied to assess the efficacy of drugs in reducing liver fat. In this study, MRI-PDFF was used to quantify LFC in study subjects before and after treatment completion. FibroScan examinations with transient elastography technology were used to assess liver fibrosis based on liver stiffness measurements (LSM) and liver steatosis using controlled attenuation parameters (CAP). A FibroScan-aspartate aminotransferase (FAST) score was used for evaluation of NAFLD.
